# The tumor suppressor gene *TRC8/RNF139 *is disrupted by a constitutional balanced translocation t(8;22)(q24.13;q11.21) in a young girl with dysgerminoma

**DOI:** 10.1186/1476-4598-8-52

**Published:** 2009-07-30

**Authors:** Stefania Gimelli, Silvana Beri, Harry A Drabkin, Claudio Gambini, Andrea Gregorio, Patrizia Fiorio, Orsetta Zuffardi, Robert M Gemmill, Roberto Giorda, Giorgio Gimelli

**Affiliations:** 1 Biologia Generale e Genetica Medica, Università di Pavia, 27100 Pavia, Italy; 2 Department of Genetic Medicine and Development, University of Geneva Medical School, and University Hospitals, 1211 Geneva, Switzerland; 3 IRCCS E. Medea, 23842 Bosisio Parini (LC), Italy; 4 Division of Hematology/Oncology, Dept of Medicine and Hollings Cancer Center, 96 Jonathan Lucas St., Medical University of S. Carolina, Charleston, SC 29425, USA; 5 U.O di Istologia ed Anatomia Patologica, Istituto G. Gaslini, 16147 Genova, Italy; 6 Laboratorio di Citogenetica, Istituto G. Gaslini, 16147 Genova, Italy; 7 Fondazione IRCCS Policlinico San Matteo, Pavia 27100, Italy

## Abstract

**Background:**

*RNF139/TRC8 *is a potential tumor suppressor gene with similarity to PTCH, a tumor suppressor implicated in basal cell carcinomas and glioblastomas. *TRC8 *has the potential to act in a novel regulatory relationship linking the cholesterol/lipid biosynthetic pathway with cellular growth control and has been identified in families with hereditary renal (RCC) and thyroid cancers. Haploinsufficiency of *TRC8 *may facilitate development of clear cell-RCC in association with *VHL *mutations, and may increase risk for other tumor types. We report a paternally inherited balanced translocation t(8;22) in a proposita with dysgerminoma.

**Methods:**

The translocation was characterized by FISH and the breakpoints cloned, sequenced, and compared. DNA isolated from normal and tumor cells was checked for abnormalities by array-CGH. Expression of genes *TRC8 *and *TSN *was tested both on dysgerminoma and in the proposita and her father.

**Results:**

The breakpoints of the translocation are located within the LCR-B low copy repeat on chromosome 22q11.21, containing the palindromic AT-rich repeat (PATRR) involved in recurrent and non-recurrent translocations, and in an AT-rich sequence inside intron 1 of the TRC8 tumor-suppressor gene at 8q24.13. *TRC8 *was strongly underexpressed in the dysgerminoma. Translin is underexpressed in the dysgerminoma compared to normal ovary.

*TRC8 *is a target of Translin (TSN), a posttranscriptional regulator of genes transcribed by the transcription factor CREM-tau in postmeiotic male germ cells.

**Conclusion:**

A role for *TRC8 *in dysgerminoma may relate to its interaction with Translin. We propose a model in which one copy of *TRC8 *is disrupted by a palindrome-mediated translocation followed by complete loss of expression through suppression, possibly mediated by miRNA.

## Background

Dysgerminomas are rare ovarian tumors most common in adolescent women, representing 5–10% of all malignant ovarian tumors in the first two decades of life. They arise from germ cells within the gonad and represent the ovarian counterpart of testicular seminoma[1]. Histologically and clinically dysgerminomas are classified as type II Germ Cell Tumors (GCT).

The female germ cells enter meiosis during intrauterine development (11–12 weeks of gestation), whereas for the male germ cells this only happens after the onset of puberty. This could explain the difference in incidence of the type II gonadal GCTs between females and males and the median age of clinical manifestation. In fact, the number of target cells (PGCs/gonocytes) for initiation is significantly lower in females compared with males [2].

*RNF139/TRC8 *(NM_007218; henceforth, *TRC8*) is a potential tumor suppressor gene (TSG) with similarity to *PTCH *[3]. Mutations in *PTCH *result in predisposition to basal cell carcinoma and medulloblastoma, while its inactivation leads to cell proliferation [4-6]. *TRC8 *was identified in a family with the constitutional translocation t(3;8)(p14.2;q24.1), and hereditary renal cell carcinoma (RCC) and thyroid cancer[3,7,8]. Recently, a second, independent, family with hereditary kidney cancer was discovered that carries a cytogenetically indistinguishable translocation and a VHL mutation. [9]. Secondary loss of the wild type *TRC8 *allele was observed in a subset of tumor cells, consistent with tumor suppressor function. TRC8 is a membrane-bound E3 ubiquitin ligase that inhibits the growth of cells in a ubiquitylation-dependent manner [10]. Interestingly, its level and stability are modulated by cholesterol and it interacts with components of the lipid homeostatic machinery, especially INSIG and the lipid-regulated transcription factors, SREBP1/2 [11]. A frequent genetic alteration in both hereditary and sporadic RCC is mutation of *VHL*, which encodes the targeting subunit of another ubiquitin ligase complex responsible for degrading hypoxia inducible factors, HIF1/2 alpha. TRC8 interacts with VHL and, in fruit flies, knockdown of either gene led to a similar embryonic phenotype [12]. Thus alterations in TRC8 have the potential to affect cellular growth control and possibly its linkage to lipid homeostasis and/or hypoxia responses.

*TRC8 *has been identified as a target of the Translin (TSN) gene in postmeiotic male germ cells, and testis is known to contain higher levels of *TRC8 *mRNA than other tissues[3]. TSN, formerly known as testis brain-RNA binding protein, is found in the cytoplasm and functions as a posttranscriptional regulator of a group of genes transcribed by the transcription factor CREM-tau. Cho *et al*.[13] identified four new TSN target mRNAs encoding diazepam-binding inhibitor-like 5, arylsulfatase A, a tetratricopeptide repeat structure-containing protein, and *TRC8*. These observations suggest an important function for *TRC8 *in germ cells. Here, we describe a paternally inherited balanced translocation t(8;22) in a proposita with dysgerminoma. The breakpoints of the translocation are located within the LCR-B low copy repeat on chromosome 22q11.21, containing the palindromic AT-rich repeat (PATRR) involved in recurrent and non-recurrent translocations and in an AT-rich sequence inside intron 1 of the TRC8 tumor-suppressor gene at 8q24.13. This translocation raises the possibility that disruption of *TRC8 *may contribute to development of the proposita's dysgerminoma.

## Methods

### Clinical report

The proband is the only one child born to healthy non-consanguineous parents with an unremarkable family history. Neither cancers nor miscarriages were reported among her relatives. She was born at term after an uneventful pregnancy. At birth, her growth parameters were normal. The neonatal period was normal. She was referred to our Institute at the age of 11 years because of persistent abdominal pain with fever and evidence of a pelvic solid mass discovered with ultrasonography in another hospital.

A total body CT scan upon admission showed a large (17 × 12 × 10 cm) inhomogeneous and necrotic mass arising from the pelvis with important displacement of the urinary tracts and bilateral hydronephrosis; a concomitant peritoneal fluid was also present in the pouch of Douglas.

No distant lesions were detected in other abdominal organs, CNS and lungs. The Tc-99 scintigraphy was negative for bone lesions. Among serum markers, only LDH (12980 U/L) (N.V. 84–362 U/L) and β-hCG (113 U/L) (N.V. <5 U/L) were abnormal for age, suggesting the hypothesis of a germinal tumor. A surgical approach was decided as first treatment. During surgery, a tumoral mass arising from the left ovary was completely resected with consensual monolateral annessectomy; a biopsy of the contra lateral ovary and a sample of peritoneal fluid were also sampled for histological evaluation.

Histological analysis confirmed a classic dysgerminoma of the ovary without evidence of tumoral cells in other tissue samples. No chemotherapy was administered after surgery according to Italian protocol (TCG 2004) for stage 1 ovarian dysgerminoma and the patient was discharged with a follow-up program. Nine months after the diagnosis the patient is healthy and shows normal β-hCG values for age.

### Hystology and Immunohistochemistry

Formalin-fixed paraffin-embedded tissue specimens were cut from paraffin blocks and stained with hematoxylin and eosin for microscopic examination. Immunohistochemical labelling was performed by a three-step indirect immunoperoxidase technique. Formalin fixed, paraffin embedded, 3 micron serial tissue sections were incubated at room temperature for 30 minutes with the antibodies reported in Table 1. Additional sections were incubated overnight at 4°C with a rabbit polyclonal antibody against the C-terminus of the p145 human c-kit protein (Oncogene, Boston, MA, USA) and with hypoxia-related antibodies: anti-HIF-2α mAb (clone ep190p, Abcam; Cambridge, UK), anti-VEGF polyclonal Ab (Santa Cruz Biotechnology; Santa Cruz, CA), anti-FGFR (clone VBS1, Chemicon; Temecula, CA), anti-VHL (clone 3F391, Abcam), anti-CA IX polyclonal Ab (Abcam), anti-COX2 polyclonal Ab (Abcam), anti-IGF1R (clone SPM138, Abcam). A monoclonal antibody anti-RNF139/TRC8 (Abcam) was used on normal fallopian tube and dysgerminoma specimens from our proposita.

**Table 1:** Antibodies

**Antibody**	**Source**
anti-MNF116 cytokeratin mAb)	(clone MNF116, Dako; Glostrup, Denmark

anti-AE1/AE3 cytokeratin mAb	(clone AE1/AE3, Dako)

anti-CD66e mAb	(clone 12-140-10, Novocastra; Newcastle, UK)

anti-EMA mAb	(clone GP1.4, Novocastra)

anti-vimentin mAb	(clone V9, Novocastra)

anti-PLAP mAb	(clone 8A9, Novocastra)

anti-beta HCG mAB	(Novocastra)

anti-NSE mAb	(clone 5E2, Novocastra)

anti-GFAP mAb	(clone GA5, Novocastra)

anti-desmin mAb	(clone DE-R-11, Novocastra)

anti-Ki 67 mAb	(clone MIB-1, Dako)

anti-CD31 mAb	(clone JC70A, Dako)

anti-CD45	(clone T29/33, Dako)

anti-CD68 mAb	(clone KP1, Dako)

anti-CD117	polyclonal Ab (Dako)

p145 human c-kit protein	(Oncogene, Boston, MA, USA)

CD30	(Clone BER-H2)Dako

S100	Polyclonal Ab, Dako

CD3	Polyclonal Ab, Dako

CD20	Clone L26, Dako

CD45	Clone T29/33, Dako

CD56	Clone CD564, Novocastra

Synaptophysin	Clone SY38, Dako

Chromogranin A	Clone DAK-A3, Dako

Sections were subsequently incubated at room temperature with anti-mouse or anti-rabbit Ig antibodies conjugated to peroxidase-labelled dextran polymer (Dako). The chromogen 3, 3-diaminobenzidine was used in the presence of hydrogen peroxide. Slides were counterstained with Mayer's haematoxylin.

Negative controls were performed using blocking serum in place of primary antibody. Positive and negative controls were run in parallel with each batch, and appropriate results were obtained. Finally, histochemical reaction of cryosectioned tumor was used to evaluate lipid deposition. Cryosections were fixed in 10% neutral buffered formalin. Samples were stained with Oil Red "O" kit (Diapath, Martinengo, Italy).

### Cytogenetics investigations

Chromosome preparations were made from cultured lymphocytes from the proposita and her parents using standard high-resolution techniques. To define the breakpoint of the translocation we used fluorescent in situ hybridization (FISH) with BAC clones spanning the chromosomal 8q24.1 and 22q11.2 regions selected according to the University of California Santa Cruz (UCSC) Human Genome Assembly (March 2006 assembly).

### FISH analysis on paraffin embedded tumoral tissue

To prepare paraffin-embedded tissue sections fixed on positively charged slides, we cut 4 – 5 μm thick paraffin sections using a microtome. Floating sections were mounted on positively charged slides. To deparaffinize specimens, slides were treated by Paraffin Pretreatment Kit (Abbott Molecular Inc., IL, USA) and treated with protease, then hybridization was performed with the appropriate Vysis protocol.

### Array – CGH analysis

Molecular karyotyping was performed using the Human Genome CGH Microarray Kits 44B and 244A (Agilent Technologies, Palo Alto, CA, USA) covering the whole genome with a resolution of ~100 kb and ~35 Kb, respectively. Briefly, 1 μg of patient and sex-matched pooled reference DNAs were processed according to the manufacturer's protocol. Fluorescence was scanned in a dual-laser scanner and the images were extracted and analyzed with Agilent Feature Extraction software (v9.5.3.1) and CGH Analytics software (v3.5.14) respectively. Changes in test DNA copy number at a specific locus are observed as the deviation of the log ratio value from a modal value of 0.

### Generation of somatic cell hybrids

Somatic cell hybrid clones were generated by fusing the HPRT-negative RJK88 Chinese hamster cell line with lymphoblastoid cell lines (LCLs) from the patient[14].

### DNA extraction and genotyping

Genomic DNA from fresh and frozen samples was extracted using a standard proteinase K digestion, followed by phenol/chloroform extraction, and resuspended in water. FFPE material from rolled sections was extracted using the QiaAmp DNA Mini Kit (Qiagen) according to manufacturer's instructions, with minor modifications. DNA quantitation was determined by spectrophotometry (NanoDrop, Thermo Scientific, Wilmington, DE). Other genomic DNAs from tissues and cell lines were extracted with DNAzol (MRC, Inc, Cincinnati, OH). Genotyping of polymorphic loci was performed by amplification with primers labeled with fluorescent probes (ABI 5-Fam, Hex and Tet) followed by analysis on an ABI 310 Genetic Analyzer (Applied Biosystems). Non-polymorphic loci were assayed by electrophoresis on agarose gels. All primers were purchased from MWG Biotech AG (Ebersberg, Germany). The sequences of all primers used are available from the authors.

Southern blot analysis was conducted on 5 μg aliquots of genomic DNA cut with SacI and PstI restriction enzymes. The DNAs were separated on 0.8% agarose/0.5× TBE gels, transferred to Hybond-N+ (GE Healthcare, Italy), hybridized to non-radioactively labelled probes (Gene Images Random-Prime DNA Labeling kit, GE Healthcare, Italy) and detected with CDP-Star detection reagent (GE Healthcare, Italy).

### Expression analysis

Total RNA was extracted from all tissues and cell lines with Trizol (Invitrogen, Milano, Italy) following manufacturer's protocols. Additional Human Tissue Total RNA was purchased from Stratagene. cDNA synthesis was performed with Ready-To-Go You-Prime First strand beads (Amersham) and random hexamers; Non-quantitative RT-PCR was performed in 25 μl reactions, using JumpStart Red ACCUTaq LA DNA polymerase (Sigma) and the following protocol: 1 min at 96°C; 30 cycles of 30 sec at 94°C/30 sec at 58°C/2 min at 68°C; 5 min at 68°C final elongation time; PCR products were analysed on 1.5% agarose TAE gels. G3PDH amplification primers and protocol were from Clontech.

Quantitative gene expression was assessed by Real-Time Quantitative PCR (RT-Q-PCR) on a 7900 HT Sequence Detection System (Applied Biosystems) using SYBR^®^Green PCR Master Mix (Applied Biosystems). Validation experiments demonstrated that amplification efficiencies of the control and all target amplicons were approximately equal (not shown); accordingly, relative quantification of DNA amount was obtained using the Comparative CT method (described in Applied Biosystems User Bulletin #2, December 11, 1997: ABI PRISM 7700 Sequence Detection System).

### *TRC8 *mutation and CpG island methylation analysis

Primer pairs were selected covering the promoter region (1 pair), exon 1 (1 pair), and the protein coding region of exon 2 (4 pairs) of the TRC8 gene. PCRs were performed in 25 μl reactions, using JumpStart Red ACCUTaq LA DNA polymerase (Sigma) and the following protocol: 30 sec at 96°C; 35 cycles of 30 sec at 94°C/30 sec at 60°C/2 min at 68°C; 10 min at 68°C final elongation time; 5% DMSO was added to promoter and exon 1 PCRs. All PCR products were analysed on 1.5% agarose TAE gels.

We also analyzed CpG methylation of the TRC8 promoter-associated CpG island. Bisulphite treatment was carried out as described by Kubota *et al*.[15]. Methylation levels were tested by sequencing and restriction mapping of the fragments amplified from bisulphite-treated DNA with TaqI restriction enzyme. The VHL gene was sequenced from tumor and normal DNA according to the methods described by Poland *et al*. [9].

## Results

### Histology and Immunohystochemistry

The left ovary measured 17 × 12 × 10 cm and its weight was 1,115 g. The cut surface was solid, partly nodular, and fleshy, with gray/rose coloration and some necrotic areas. Histologically, the tumor was composed of uniform cells consistent with a diagnosis of dysgerminoma. No metastases were demonstrated microscopically in any of the retroperitoneal lymph nodes examined. The right ovary was apparently normal with a light elongated shape.

**Figure 1:**
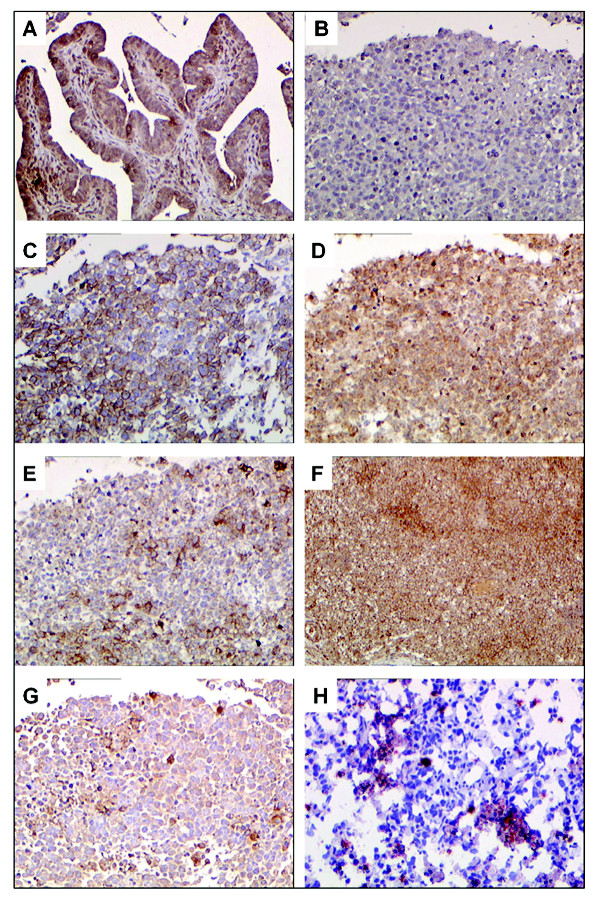
**Immunohistochemical analysis of antigens in dysgerminoma**. (A) normal Fallopian tube; (B-H) dysgerminoma. (A) intense TRC8 immunoperoxidase expression in control tissue. (B) negative immunoperoxidase TRC8 reaction in dysgerminoma; C, CD117 strong membrane staining in dysgerminoma; D, diffuse and intense VHL expression; E, FGFR cytoplasmic expression; F, diffuse and intense CAIX expression; G, HIF2alpha nuclear and cytoplasmic expression in sporadic cells; H, moderate lipid deposition detected by Oil Red O. Original magnification A-H ×100.

The tumor showed strong positive staining (with membrane enhancement) for PLAP, CD117 and CD68, focal positivity for MNF116, while the balance of antibodies were negative. TRC8 was positive in normal Fallopian tube but negative in the dysgerminoma (Fig. 1A, B). Expression profiles of hypoxia-related tissue factors were as follows: HIF-2α and FGFR expression was detectable only in a minority of cells, while VHL and CA IX were consistently expressed in the context of CD117 positive vital tumor tissue (Fig. 1C–G); in the same areas, immunolabeling with the C-terminus of the p145 human c-kit protein was negative. Histochemical reaction with Oil Red "O" on cryosectioned tissue tumor showed a moderate deposition of lipids in selected areas (Fig 1H).

### Cytogenetic analysis and mapping of the translocation breakpoints

**Figure 2:**
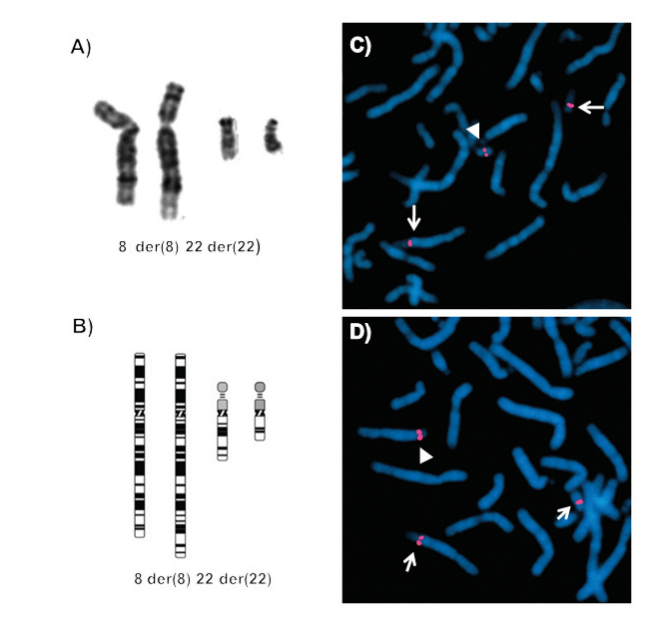
**Cytogenetics and FISH results from the proposita's lymphocytes, A, cut-out and B, idiogram of the normal (left) and derivative (right) chromosomes 8 and 22**. C, FISH with BAC RP11-562F10 (red signals) spanning the breakpoint on the long arm of chromosome 22. D, FISH with BAC RP11-158K1 demonstrates that the chromosome 8 breakpoint is within this clone. Arrowheads indicate the signal on the normal chromosome while arrows mark derivative chromosomes.

Giemsa staining identified a chromosomal translocation (8;22)(q24.13;q11.21) in the proband (Fig. 2A, B). The translocation was also present in her apparently normal father, while her mother had a normal karyotype. No additional relative in the paternal lineage was available for testing. FISH analysis using genomic clones localized the translocation breakpoints to within RP11-562F10 (GenBank Accession No. AZ537723) at 22q11.21 (Fig. 2C) and RP11-158K1 (GenBank Accession No. AF313041) at 8q24.13 (Fig. 2D). Five known genes have been mapped to the RP11-158K1 clone (*TRMT12, TRC8, TATDN1, NDUFB9, MTSS1*) and three to RP11-562F10 (*ZNF74, SCARF2, KLHL22*). This latter BAC clone also contains two segmental duplications, DC3315 and DC3316.

**Figure 3:**
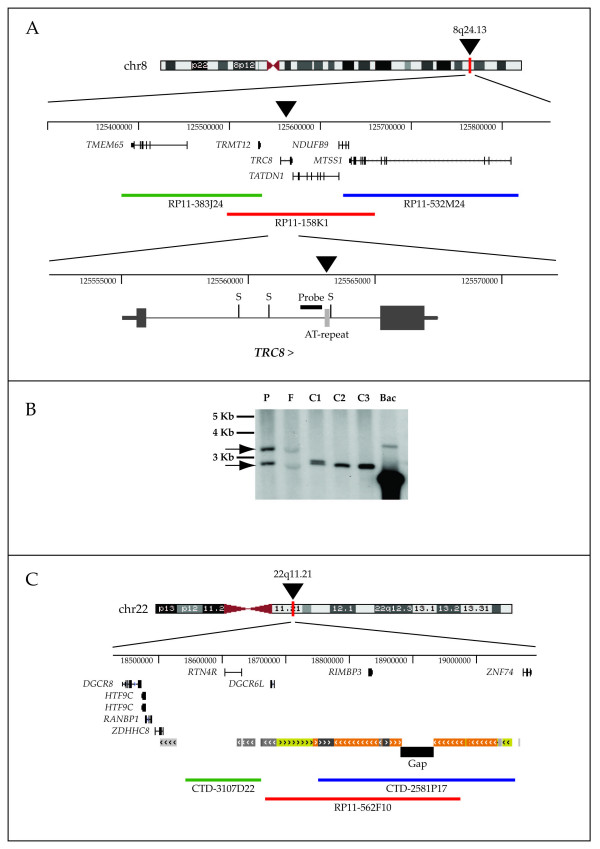
**Map of the translocated regions**. (A) schematic of the genomic region rearranged on chromosome 8 showing the translocation breakpoint (black arrowhead), the BAC clones used to refine the cytogenetic characterization (colored bars), and the molecular localization of the breakpoint to an AT-rich simple repeat (light grey box) in intron 1 of the *TRC8 *gene. The position of the *SacI *restriction sites (S) and of the probe used for Southern analysis are indicated. (B), Southern blot showing *SacI*-cleaved fragments of the first intron containing the AT-rich repeat in the proband (P), her father (F), several normal controls (C1–3) and the BAC RP11-158K1 (Bac). Positions of the proband's alleles (arrows) and two molecular weight markers are shown. (C) Localization of the chromosome 22 breakpoint, contained within BAC RP11-562F10.

To further characterize the breakpoints, we generated somatic cell hybrids from the lymphoblastoid cell line of the proposita. From 100 hybrid clones, one contained only a normal chromosome 8, six retained a normal chromosome 22, seven retained the der(8) and four retained the der(22) chromosome. Based on the UCSC (March 2006 freeze) map, we typed the hybrids for informative chromosome 8 markers (see Table 2) and localized the breakpoint to a 700 bp region in intron 1 of the TCR8 gene. This region contains an *AluSp *and a 180 bp (AT)n simple repeat (Fig. 3A, B).

**Table 2:** Genomic typing of somatic cell hybrid clones with chromosome 8 and 22 markers.

	**Locus**	**Position (Kb)**	**Genomic**	**Chr. 8**	**Chr. 22**	**der(8)**	**der(22)**
**Chromosome 8**	D8S1784	106172	287	287		287	

	D8S514	123811	221/223	223		221	

	D8S1832	125517	180/186	180		186	

	RH1927	125534	+	+		+	

	RNF139 E1	125556	+	+		+	

	Chr8-2-1	125559	+	+		+	

	Chr8-2-2	125562	+	+		+	

	Chr8-3-1	125563	+	+		+	

	Chr8-3-2	125564	+	+			+

	Chr8-2-3	125565	+	+			+

	RNF139 E2	125567	+	+			+

	TATDN1 E10	125570	+	+			+

	TATDN1 E6	125590	+	+			+

	NDUFB9 E2	125625	+	+			+

	NDUFB9 E4	125631	+	+			+

	MTSS1 E14	125635	+	+			+

	MTSS1 E13	125637	+	+			+

	MTSS1 E11	125639	+	+			+

	D8S1799	125646	234	234			234

	D8S272	137804	241/255	255			241

**Chromosome 22**	D22S427	16971	100		100		100

	RH78249	18480	+		+		+

	RH57884	18609	+		+		+

	RH102235	19092	+		+	+	

	RH93744	19126	+		+	+	

	D22S280	31539	210/212		210	212	

The 8q24 breakpoint region was refractory to amplification (not shown). We examined the repeat-rich segment by digestion with *SacI *followed by Southern blot analysis and observed two bands both in the proband and her father; the larger shared fragment presumably contains the rearrangement's breakpoint. One of the normal control samples also contained two alleles; in all cases, the fragments were several hundred bases larger than in the RP11-158K1 clone (Fig. 3B), consistent with polymorphism of the AT-rich VNTR.

On chromosome 22, the breakpoint was localized to the LCR-B [16]/LCR22-3a [17] which is the same region involved in the recurrent t(11;22)(q23;q11.2) [16,18,19] as well as t(17;22)(q11.2;q11.2) and several other translocations [17] (Fig. 3C). Since most of the 22q11 breakpoints involved in these translocations occur at the center of PATRR22, we postulated that this was also the most likely breakpoint in our case, but the breakpoint junctions could not be amplified using the method described by Gotter *et al*[20].

**Figure 4:**
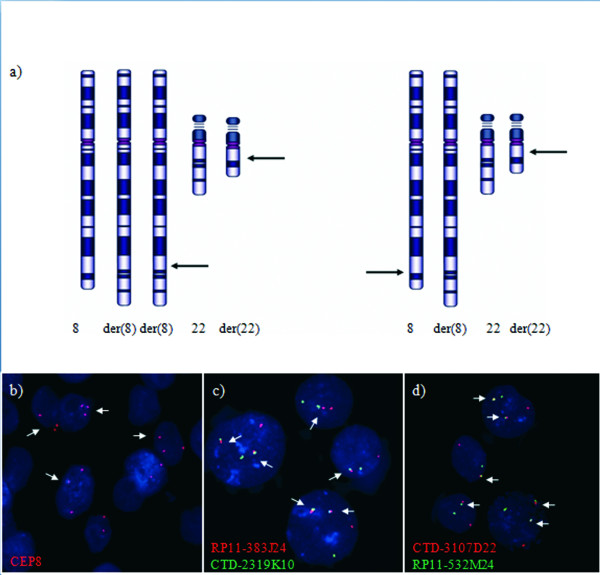
**Interphase FISH on deparaffinized sections from dysgerminoma**. a) schematic representation of the results of the interphase FISH. b) Nuclei show two or three red signals (CEP8) indicating the presence of nuclei with two or three chromosomes 8, respectively.c) Dual color hybridization with BAC RP11-383J24 (red signal) and BAC CTD-2319K10 (green signal) indicate the presence of a normal chromosome 8 and a normal chromosome 22 (single signals); nuclei show one or two derivative chromosome 8 (arrows). d) Dual color hybridization with BAC CTD-3107D22 (red signal) and BAC RP11-532M24 (green signal) indicate the presence of a normal chromosome 8 and a normal chromosome 22 (single signals) and one or two derivative chromosome 22 (arrows).

To investigate the presence of the translocated chromosomes in the tumoral tissue, we performed a dual colour hybridization experiment on deparaffinized slices from the dysgerminoma (summarized in Fig. 4). We used a probe specific for the centromere of chromosome 8 (CEP8; Vysis) to visualize its copy number in single cells. To detect the normal and derivative chromosomes 8 and 22 we used dual FISH with BACs RP11-383J24 (chromosome 8) and CTD-2319K10 (chromosome 22) both adjacent to the breakpoints, to unambiguously identify the derivative 8 (Fig. 4C). Dual FISH was also performed with BACs CTD-3107D22 (chromosome 22) and RP11-532M24 (chromosome 8) to detect the derivative 22 (Fig. 4D). This experiment revealed that ~40% of tumor cells contained two copies of the derivative 8 while while the remaining 60% contained one copy. Similar results were obtained for the derivative 22; all cells also contained one normal chromosome 8 and one normal chromosome 22.

### Array-CGH results

**Figure 5:**
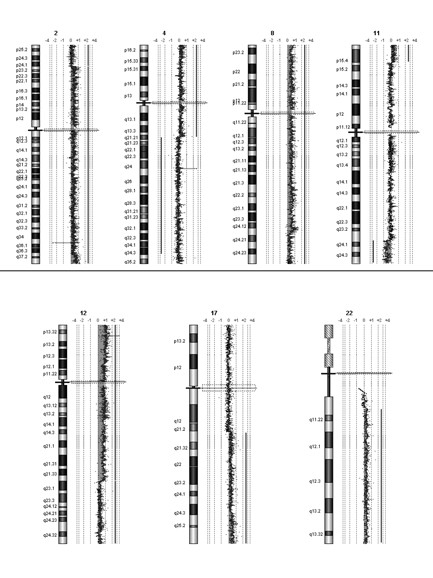
**Array comparative genome hybridization profiles of the chromosomes involved in rearrangements in the dysgerminoma**.

We carried out array-CGH analysis on the dysgerminoma's DNA to map all chromosomal imbalances at ~100 kb average resolution. Non-mosaic gain of an entire chromosome was detected for chromosomes 7 and 15, while mosaicism for a whole-chromosome gain was detected for chromosomes 1, 6, 16, 19, 20, and 21 (not shown). A structural chromosomal imbalance was detected for chromosomes 2, 4, 8, 11, 12, 17, and 22, both as gain or loss (see Table 3). Array comparative hybridization profiles of the abnormal chromosomes are shown in Fig. 5. Array-CGH analysis with 8.9 KB overall median probe spacing, performed on the genomic DNA of our proband and her parents, excluded a concomitant deletion or duplication.

**Table 3:** List of the imbalances found in the analysis of the Dysgerminoma.

**Chr**	**Chromosome Band**	Gain/Loss*	**Position**	**Size (Mb)**	**Notes**
Chr 2	2p24.1 → 2q37.1	enh	Chr2:24,134,000..233,241,000	209.10	mosaic 30%

Chr 2	2q37.1 → qtel	enh	Chr2:233,241,000..242,951,149	9.71	*More copies*

Chr 4	4q21.21 → qtel	dim	Chr4:80,599,000..191,273,063	110.70	mosaic 30%

Chr 8	8ptel → q24.13	enh	Chr8:1..125,719,000	125.70	

Chr 8	8q24.13 → qtel	enh	Chr8:125,719,000..146,274,826	20.56	mosaic 30%

Chr 11	11p15.3 → 11q23.3	dim	Chr11:11,570,000..119,487,000	107.90	mosaic 30%

Chr 11	11q23.3 → qtel	dim	Chr11:119,487,000..134,452,384	14.97	

Chr 12	12ptel → q23.1	enh	Chr12:1..94,196,000	94.20	*More copies*

Chr 12	12q23.1 → qtel	enh	Chr12:94,196,000..132,349,534	38.15	

Chr 17	17q21.3 → qtel	enh	Chr17:38,922,000..78,774,742	39.85	mosaic 30%

Regions interested by the deletions/duplications/triplications are reported together with their mapping position and cytogenetic bands (hg 17/Build 35, May 2004), The size of each imbalance is always related to the platform resolution having 100 kb average distribution of the clones along the genome. The size of the intervening regions is shown, although it should be considered that breakpoints may fall within a segment of 100 kb (average) proximal and distal to the starting and ending clones. * Enh = enhanced log2 ratio (gain); dim = diminished log2 ratio (loss).

### VHL gene mutations

The proband was heterozygous for multiple *VHL *SNPs in her blood DNA, while her father was homozygous for all of them. We amplified by long-range PCR and sequenced all exons of the *VHL *gene on 3p25.3 in both directions from tumor DNA, finding no evidence of mutations or loss of heterozygosity.

### Expression analysis

**Figure 6:**
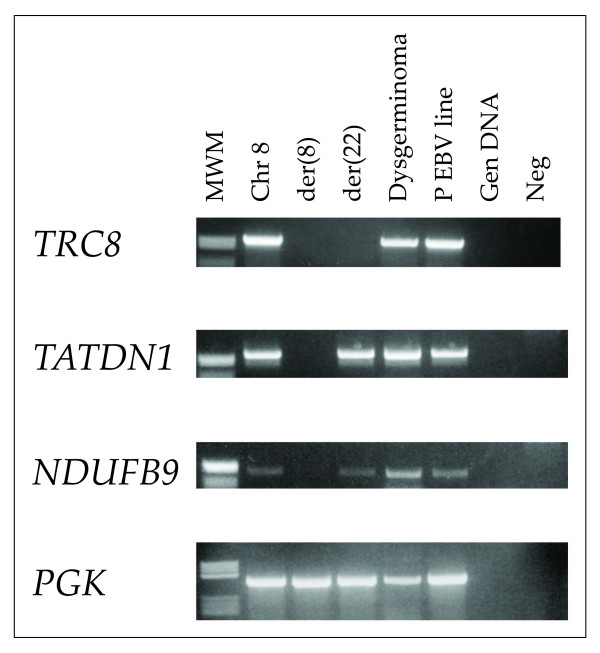
**Non-quantitative RT-PCR analysis for expression of *TRC8*, *TATDN1*, *NDUFB9 *in cell lines and dysgerminoma**. RNA isolated from indicated hybrids and proband's nomal or tumor tissues was reverse transcribed and used for PCR amplification with the indicated gene-specific primers. The housekeeping gene *PGK *was used as a control; MWM, molecular weight marker V (Roche); Neg, no DNA control.

Non-quantitative RT-PCR analysis of *TRC8 *showed that the gene was expressed in the Epstein-Barr Virus (EBV) line and in the hybrid clone containing a normal chromosome 8, but not in hybrids carrying the der(8) or der(22) chromosomes (Fig. 6). In contrast, the neighbouring genes *TATDN1 *and *NDUFB1 *were expressed in the der(22) hybrid. Expression of all three genes was detectable in the dysgerminoma.

**Figure 7:**
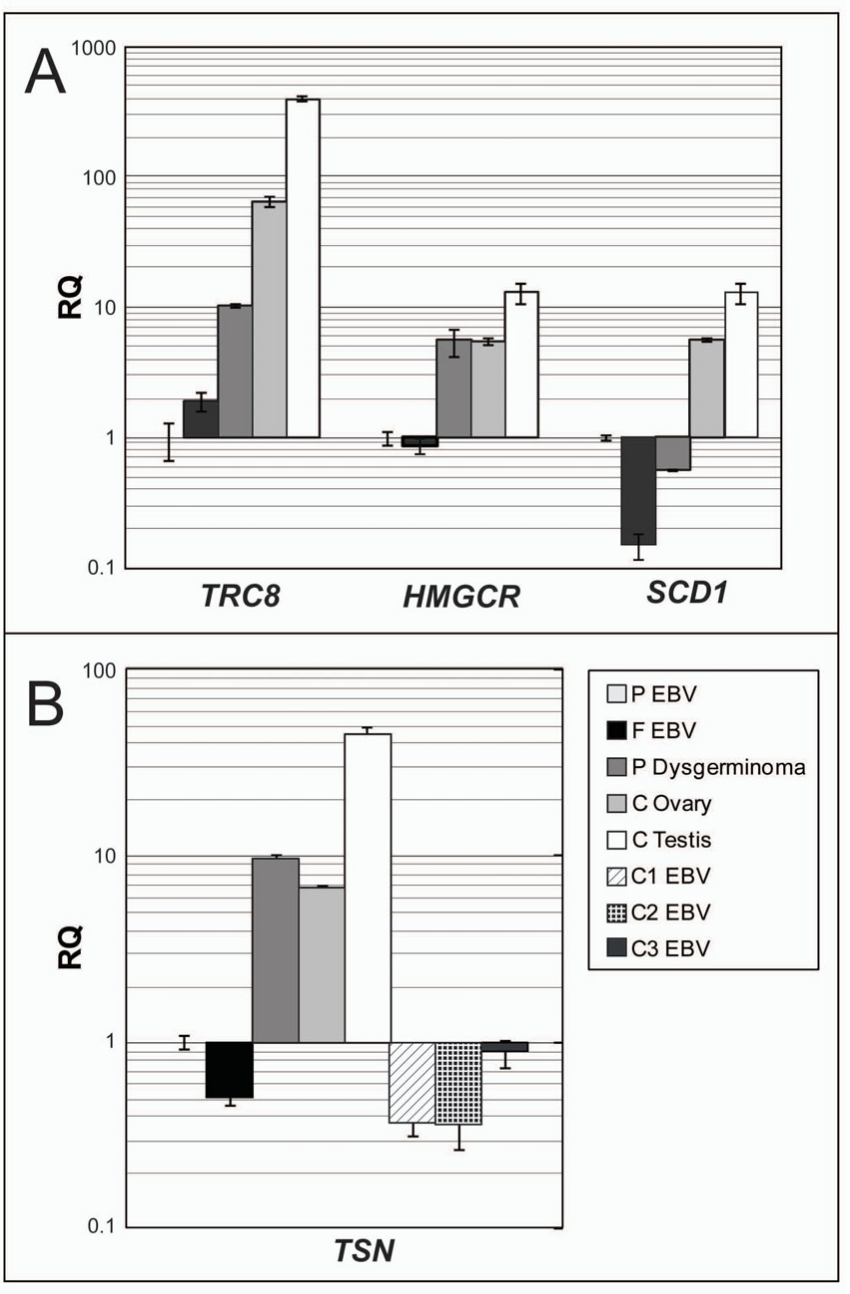
**A, Quantitative RT-PCR analysis of *TRC8*, *HMGCR*, and *SCD1 *expression in the proband's dysgerminoma, in normal tissues and in EBV lines from the proband (P) and her father (F)**. The housekeeping gene *PGK *was used as endogenous control, while proband's EBV line was the reference tissue. B, Expression analysis of translin (*TSN*) in tissues and EBV-transformed cell lines. *PGK *was again used as endogenous control. Relative quantity (RQ) is shown on a logarithmic scale.

Using Real-Time RT-PCR, we quantitated expression of *TRC8 *in normal human ovary and testis, in the dysgerminoma, in EBV lines from the proband and her father, and in three additional EBV lines from normal subjects. Ovary and testis RNA come from pooled samples (ovary: 15 caucasian females, aged 20–60; testis: 39 caucasian males, aged 14–64) and consequently can be considered "averaged" tissue samples. We also analyzed the expression of *HMGCR *and *SCD1*, two *SREBP *target genes potentially down-regulated by *TRC8 *in RCC cells, and of the housekeeping gene *PGK *(Fig. 7A). Compared to normal ovary, *TRC8 *was expressed approximately 10 times less in the dysgerminoma. In contrast, it was expressed 4-fold more in testis. All lymphoblastoid lines expressed *TRC8 *at very low levels, and there was no difference in expression between the subjects carrying the translocation and the control lines. Because of low *TRC8 *expression in the tumor, we anticipated that SREBP target genes, including *HMGCR *and *SCD1 *might be overexpressed. In contrast, we observed that *SCD1 *was strongly underexpressed while *HMGCR *was not affected in the dysgerminoma compared to normal ovary. Perhaps the complex regulation of each gene makes it difficult to discern the effect of reduced TRC8, or the whole ovary is not a good control tissue for germ cell expression. In addition, regulation of lipid homeostatic genes by *TRC8 *may be specific for some tissues or tumors. The level of *PGK *expression was comparable in all tissues.

We quantified expression of the *TSN *gene encoding translin, a protein involved in post-transcriptional regulation of a number of genes, including *TRC8*, during spermatogenesis (Fig. 7B). *TSN *was expressed approximately 3 times more in the testis than in normal ovary, while it was expressed approximately two times less in the dysgerminoma. Expression was homogeneously lower in all EBV lines. Due to the rarity of dysgerminomas, our findings are limited to the one sample we were able to collect and analyze; whether or not these expression levels are characteristic of dysgerminomas or unique to this translocation case is unknown.

### Analysis of tumor-derived TRC8 for genetic and epigenetic changes

Genomic DNA sequences, including the *TRC8 *promoter and exons 1 and 2, were examined from both the proband's EBV line and the dysgerminoma. There were no differences between the two sequences, and both were identical to the human reference sequence (NC_000008, from 125556189 to 125570040). Sequences of the *TRC8 *transcript reverse-transcribed from the EBV line and dysgerminoma were also identical to the database. Lastly, we examined promoter methylation patterns of *TRC8*, and found it was completely unmethylated in both tissues (not shown)

## Discussion

We report a girl who developed a dysgerminoma at the age of 10 years and carries a balanced translocation t(8;22)(q24.13;q11.21). The breakpoints of our proposita may be identical to those recently described by Gotter *et al*., [20]. In that case, the proband showed an unbalanced translocation with a supernumerary der(22) derived as a result of 3:1 meiotic malsegregation of the paternal balanced translocation 46, XY, t(8;22)(q24.13;q11.21). The proband showed an abnormal phenotype, while his father was apparently normal. Interestingly, a breakpoint within the first intron of the *TRC8 *gene has been described as an inherited rearrangement associated with renal cell carcinoma in two unrelated families[3,9,21,22]. In our case, we have localized the breakpoint to a 700 bp region in intron 1 of the *TCR8 *gene, containing an *AluSp *and a 180 bp (AT)n simple repeat. The breakpoint is probably positioned, as in the family described by Gotter *et al*.[20], in a palindrome within the simple repeat. In our case and in Gotter *et al*.[20], the breakpoint on chromosome 22 lies within the PATRR on 22q11. The analysis of many unrelated t(11;22)(q23;q11) cases revealed that the breakpoints occur within palindromic PATRRs on 11q23 and 22q11 (PATRR11 and PATRR22). The majority of the breakpoints, including the case described by Gotter *et al*.[20], are localized to the centre of the PATRRs, suggesting that the palindrome mid-point is susceptible to double-strand breaks (DSBs); this would occur as the result of a cruciform extrusion promoting the DSBs that initiate stabilizing rearrangements or recombination events[23]. The PATRR22 appears to be highly unstable in the human genome[24]. Palindrome-mediated genomic instability contributes to a variety of genome rearrangements including not only constitutional translocations, but also large germ line deletions[25]. Cancer-related gross chromosomal rearrangements and genomic amplification are also reported to be associated with palindromic DNA[26,27]. Recent studies have produced a detailed map showing that palindromes tend to cluster at specific regions, some of which undergo gene amplification[28]. Individual tumors seem to have a non-random distribution of palindromes in their genomes, and a subset of palindromic loci is associated with gene amplification. Inverted repeats or palindromes are known to generate hairpins or cruciform structures, facilitated by intrastrand pairing of complementary single-strand DNA sequences assembled in the lagging strand during DNA replication. A double-strand break (DSB) introduced in these hairpins can result in deletions or in either inter- or intrachromosomal recombination events mediated by the homologous recombination machinery[26,28].

*TRC8 *encodes an endoplasmic reticulum-resident E3-ubiquitin ligase with multiple transmembrane segments. TRC8 contains a C-terminal RING-H2 domain shown to catalyze *in vitro *ubiquitylation reactions[10,11]. In *Drosophila *and mammalian cells, over-expressed TRC8 suppressed growth[11,12,29]. In HEK293 cells, TRC8 induced G2/M arrest and accumulation of sub-G1 cells indicative of apoptosis[11]. These effects were dependent upon an ubiquitylation-competent RING domain, suggesting that specific substrates were critical to growth inhibition. Tumor formation in a nude mouse model was also inhibited by TRC8 in a RING-dependent manner. TRC8 contains a putative sterol-sensing domain (SSD) which in other proteins confers sensitivity to sterols, affecting either stability or trafficking between membrane compartments[3]. Moreover, knock-down of endogenous TRC8 increased levels of the lipid homeostasis transcription factors, the SREBPs and their target genes, including enzymes of cholesterol and lipid metabolism [11]. However, these effects were only evident in cells simultaneously depleted of sterols, since cholesterol-replete cells contain very low amounts of TRC8. Importantly, expression of activated SREBP-1 partially restored the growth of TRC8-inhibited cells [11], indicating that changes in lipid regulation underlie some of the growth inhibitory actions of this tumor suppressor. Since these data suggested that TRC8 activity linked growth control to the cholesterol/lipid homeostasis pathway, we analyzed two SREBP target genes, *HMGCR *and *SCD1*, in control and tumor tissue (Fig. 6). However, expression levels were depressed compared to normal ovarian tissues. This could be due to lack of proper control tissues (germ cells as opposed to whole ovary) or perhaps more likely to the presence of plentiful lipids, since the effects of *TRC8 *loss are most readily observed following sterol-depletion [11].

Interestingly, *TRC8 *expression appeared down-regulated in tumor tissue (Fig. 6) while TRC8 protein was not detectable in tumor tissue by IHC (Fig. 1). This was despite the absence of any evidence for loss, genetic alteration or promoter methylation of the remaining *TRC8 *allele. We surmise that additional mechanisms reducing *TRC8 *expression may be involved in this dysgerminoma. For example, the 3' UTR of *TRC8 *contains target sites for multiple microRNAs (miR), several of which are highly conserved among human, mouse, rat, dog and chicken (miR-218, -101, -27, -128, -375). In addition, the coding sites for some of these miRs are found on the aneuploid chromosomes observed in this tumor (i.e., miR-218-1 is on chromosome 4). It is possible that miRNAs are mediating the additional loss of expression for this gene. Recently, the stabilization of specific miRNAs by Translin in male germ cells has been reported[30].

In our patient Translin (TSN), a protein involved in post-transcriptional regulation of *TRC8 *and of a number of genes during spermatogenesis, is expressed two times less in the dysgerminoma than in normal ovary.

Translin, formerly known as testis brain-RNA binding protein (TB-RBP), is both a DNA-binding and RNA-binding protein. As a DNA binding protein, it interacts with the breakpoint junctions of chromosomal translocations[31,32]. Translin also mediates intracellular and intercellular mRNA transport, possibly controlling the temporal and spatial translation of specific mRNAs in postmeiotic germ cells [33-35]. Kasai *et al*. [36] have proposed that human Translin may play a role in DNA recombination, repair, and metabolism, but deficiencies in somatic recombination or DNA repair are not readily detectable in mice lacking *Tsn *[37]. However, the marked increase in apoptosis of spermatocytes in *Tsn*-null mice suggests a role for *TSN *during meiosis, perhaps functioning as a post-transcriptional regulator in spermatocytes [37]. Recently, Cho *et al*. [13] identified four meiotic mRNA targets of Translin, one of which is *TRC8*. They hypothesized that disruption of the precise regulation of *TRC8 *may cause the growth retardation and the increased apoptosis in pachytene spermatocytes observed in *Tsn*-null mice [37]. To our knowledge, there are no studies of the role of the *TSN *gene in female meiosis.

*TRC8 *is a potent TSG involved in different tissue-specific pathways. Its haploinsufficiency may facilitate the development of CC-RCC in association with *VHL *mutations, or otherwise lead to increased risk for other types of tumor. Any role in dysgeminoma may relate to its interaction with translin. We envision a model whereby one copy of TRC8 is disrupted by palindrome-mediated translocation (step 1), then, in a second step, translin, miRNA, or another yet undiscovered mechanism leads to further loss of *TCR8 *expression, setting the stage for deregulated proliferation.

## Abbreviations

(RNF139): Ring Finger Protein 139; (TRC8): Translocation in Renal Carcinoma on chromosome 8; (PGC): Primordial Germ Cell; (BAC): Bacterial Artificial Chromosome; (array-CGH): Comparative Genomic Hybridization; (IHC): Immunohystochemistry.

## Competing interests

The authors declare that they have no competing interests.

## Authors' contributions

RMG, RG, GG, OZ, and CG contributed to the writing of this paper. Experimental work was done by SG, SB, HAD, PF and AG. All authors read and approved the final manuscript.
